# Evaluation of Functional Outcome Following Hybrid External Fixation in The Management of Schatzkers Type V and VI Tibial Plateau Fractures - A Prospective Study of 30 Patients

**DOI:** 10.5704/MOJ.2103.008

**Published:** 2021-03

**Authors:** Y Subash

**Affiliations:** Department of Orthopaedics, Saveetha University Saveetha Medical College and Hospital, Chennai, India

**Keywords:** tibial plateau, schatzker, hybrid fixation, rasmussen

## Abstract

**Introduction:**

Fractures of the proximal tibia are high velocity injuries often associated with soft tissue compromise especially in the type V and VI fracture patterns. Dual plating is the preferred treatment option for these injuries but not in a setting where there is extensive soft tissue injury, as this can lead to problems with wound healing. The aim of this study was to evaluate the functional outcome following hybrid external fixation in the management of Schatzkers type V and VI fractures.

**Materials and Methods:**

A total of 30 patients with type V and VI proximal tibial fractures who presented between January 2012 to January 2015 were managed with hybrid external fixation and were followed-up for a period of 3 years.

**Results:**

The mean age of the patients was 42.26 years with the left knee being more commonly affected. Schatzkers type V was the more common fracture type seen. The mean time to union was 12.06 weeks and the average range of motion achieved was 0 to 100°. The mean Rasmussens functional score was 25.4 at last follow-up and we had excellent results in 5 patients and good results in 22 patients.

**Conclusion:**

Through this study, we conclude that the hybrid external fixation is an excellent option in the type V and VI fractures with extensive soft tissue compromise. It is easy to apply, facilitates early mobilisation of the joint and gives good functional results.

## Introduction

Fractures of the proximal tibia are high velocity injuries which are quite difficult to manage due to the intraarticular nature of the fracture as well as depression and comminution seen in certain fracture patterns. They are often caused by injuries such as road traffic accidents and fall from height. In the elderly age group, they are usually caused by trivial injuries such as a slip and fall due to poor bone stock with advancing age^1,2^. They are brought about by a combination of axial loading with angular forces resulting in impaction and comminution of the anterior surface and the metaphysis. Schatzkers type one fractures are the most common type seen while type V and VI account for 15-30% of fractures^3,4^. These fractures are often associated with soft tissue compromise such as deep abrasions, compound injuries and blister formation due to extensive edema. They also have comminution at the fracture site along with ligamentous injuries, which lead to instability and are often difficult to manage. The management options available for these fractures are closed reduction and casting, unilateral external fixation, bicolumnar plating and the hybrid external fixation system. Closed reduction and casting is not advised in adults as it would be difficult to maintain reduction in a cast and could lead to complications such as malunion. This treatment option would also require a long period of immobilisation, which could end up in knee stiffness, and is only reserved in patients who are medically unfit for any surgical procedure. A unilateral external fixator is ideally used for temporary immobilisation of the fracture and in compound injuries till the condition of the soft tissues improve to facilitate a definitive internal fixation of the fracture. A longer period of immobilisation with a knee spanning external fixator can cause problems such as knee stiffness with decreased range of motion of the affected knee. The ideal treatment option would be the dual plating either through a midline incision or through two separate incisions. This option, however, requires extensive dissection leading to problems with wound healing related to decreased vascularity such as skin and muscle necrosis, as well as having increased risk of infection^5,6,7,8^.

The hybrid external fixator combines an Ilizarov ring with a standard AO frame and can be used in compound injuries as well as in fractures with extensive soft tissue compromise as a definitive fixation option. The fixator acts on the principle of ligamentotaxis to reduce the fracture and to maintain the reduction as well. It provides a stable and definitive fixation and since it does not span the joint, the knee can be actively mobilised from day one to bring about a good range of motion of the knee joint and to promote cartilage regeneration and remodeling. In cases of fractures with impaction, small incisions can be made to elevate them and the fixation can also be supplemented with K wires, cannulated cancellous screws, or mini plates. Early weight bearing in a few weeks after the fracture starts consolidating can also be done with the hybrid fixator. The aim of this study was to evaluate the role of the hybrid external fixator in the management of Schatzkers type V and VI fractures, and to assess the functional and radiological outcomes using Rasmussens grading system.

## Material and Method

A total of 30 patients with Schatzkers type V and VI tibial plateau fractures who presented between January 2012 to January 2015 were treated with the hybrid external fixator and were followed-up for a period of 3 years. This study was undertaken after obtaining approval from the ethical committee of our institution. All skeletally mature patients with Schatzkers type V and VI fractures willing for surgery and follow-up were included in our study while Schatzkers types one to four fractures, patients with ipsilateral lower limb fractures, floating knee and patients with neurovascular injury were excluded. At the time of admission, all patients were assessed clinically and radiologically. At first examination, the neurovascular status was first assessed and documented. The extent of soft tissue compromise was noted and documented as well. In patients with gross swelling of the knee, ice pack application and limb elevation with either pillows or a Bohler braun splint was done immediately. The patients were then subjected to radiographs of the affected knee with the leg, which included AP, lateral as well as oblique views. In cases of fractures with extensive communition, CT scans were taken for pre-operative planning for surgery. All fractures were classified by the Schatzkers classification and the fracture type was documented in the case records.

Routine blood investigations were then sent for and the patients were worked up for the surgical procedure after obtaining the necessary medical and anaesthetic clearance. The patients were then taken up for surgery after obtaining proper informed and written consent. The procedures were performed under spinal anaesthesia under fluoroscopic guidance. Intravenous cephazolin was given at the time of induction of anaesthesia and was continued for three days post-operatively. The fractures were assessed under fluoroscopic guidance and initially traction was given to restore length, followed by a valgus or a varus stress based on the fracture pattern and displacement. In a few cases 2mm K wires were used for the provisional fracture fixation especially in communited fracture patterns. Then a 1.8mm wire was passed from lateral to the medial aspect at a distance of 15mm from the joint surface to prevent septic arthritis from occurring. The wire was then tensioned in the standard manner and a 5/8th ring was attached to it. The second wire was passed from an anterolateral to posteromedial direction and care was taken to ensure that there was a throw of at least 60° from the first wire to provide a stable construct followed by tensioning of the wire. Two 5mm Schantz pins were then placed on the anteromedial border of the tibia and the Ilizarov ring and the Schantz pins were then connected with 250mm tubular rods and universal clamps using a hybrid clamp. Final fracture reduction was checked in both AP and lateral views and confirmed to be anatomic. In three of our patients, there was depression in the lateral condyle, hence a mini incision was made over the anterior aspect of tibia and the fragment was elevated with a bone tap and fixed with a K wire. None of the cases needed supplementation with either cancellous screws or mini plates in our series. Bone grafting consent was taken from the patients prior to the surgical procedure but it was not performed as there were no large bone defects which needed grafting.

Out of the 30 patients in our study, 8 were operated on the first day and the remaining were operated on the 2nd or 3rd day. Twenty-three fractures in our series were closed injuries with 11 of them were associated with significant soft tissue damage and blister formation. The blisters were either punctured or aspirated, ice compression and limb elevation was given, and the patients were taken up for surgery on day 2 or 3. Seven patients had Gustillo and Anderson grade 2 and 3 compound injuries for which wound debridement was done followed by the hybrid external fixator application. Two patients had a grade 3 compound injury while five patients had a grade 2 injury. Out of the seven compound fractures, four were Schatztkers type VI and three were type V. The wound was primarily sutured in six patients while one patient with a grade 3B compound fracture required a flap cover. Post-operatively all patients were made to sit up in bed on the same day of surgery and the knee and ankle were actively mobilised. Patients were then started on quadriceps exercises. They were mobilised on the 1st post-operative day with non-weight bearing walking with either a crutch or a walking frame support. Intravenous antibiotics were continued for three days post-operatively. Wound inspections in cases where mini incisions were made was done on the 3rd and 5th postop day and sutures were removed on the 12th day.

**Fig. 1:**
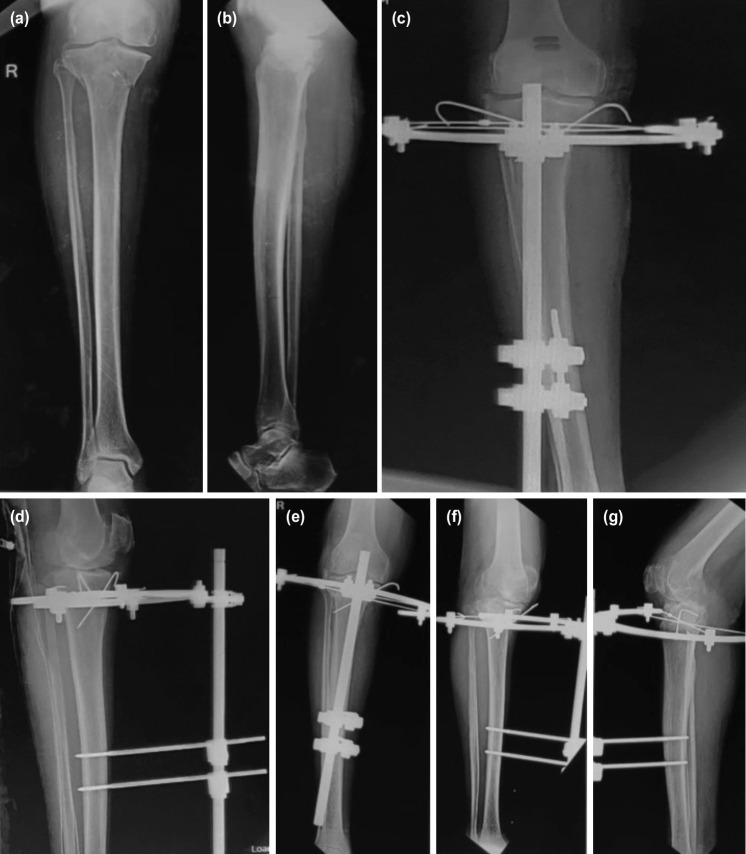
(a,b) Showing the pre-operative radiograph. (c) Showing the immediate post-operative radiograph AP view. (d) Showing the immediate post-operative lateral radiograph. (e,f,g) Showing complete union at the fracture site at three months follow-up.

**Fig. 2:**
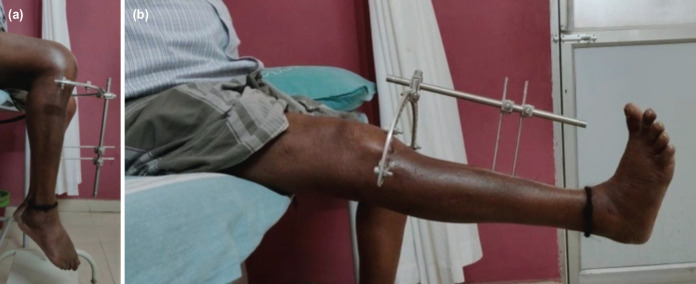
(a) Showing good range of knee flexion. (b) Showing complete extension at the knee joint.

The patients were taught how to clean the pin tracts themselves and were asked to do it daily. Post-operative radiographs were taken to assess the quality of reduction and restoration of the joint line. The patients were then discharged and were asked to review at one, three and six months and at yearly intervals thereafter where serial radiographs were taken and the progression of union was noted. Functional and radiological assessment was done using the Rasmussens scoring system and the data obtained were documented in the case records^9^. Clinical evidence of fracture union was taken to be lack of tenderness at the fracture site and absence of pain on weight bearing while radiologically it was bridging of at least three cortices in AP and lateral projection radiographs (Fig. 1,2). The hybrid fixator was removed after union at the fracture site which was at an average of 12.06 weeks. Associated ligamentous injuries were not addressed primarily and were dealt with after union of the tibial plateau fractures. Three patients had collateral ligament injuries while one patient had an isolated anterior cruciate ligament and four patients had multiple ligamentous injuries which were addressed accordingly after fracture union. The data collected was analysed using IBM SPSS Version 22.0. [Armonk, NY: IBM Corp]. Chi square test was used in the comparison of categorical variables. A P value of less than 0.05 was considered to be statistically significant.

## Results

Mean age of the patients was 42.26 years ranging from 24 to 59 years. There was a male preponderance seen in our study with the left side being more commonly affected. Schatzkers type V was the most common type seen accounting for 19 cases Table I. The time from presentation to surgery ranged from one to three days. The mean operative time was 51.33 minutes, ranging from 41 to 62 minutes. The average time to weight bearing was 8 weeks, ranging from 6 to 10 weeks. The mean duration of stay in the hospital was 12 days, ranging from 10 to 18 days. The mean time to fracture union was 12.06 weeks, ranging from 10 to 15 weeks. The average range of motion of knee achieved was 0 to 100° with one patient having an extensor lag of 10° and the average ankle range of motion was 0 to 20° dorsiflexion and 0 to 35° plantar flexion. The mean Rasmussens functional score at the time of the last follow-up was 25.4, ranging from 21 to 29 Table II. According to Rasmussens functional grading we had excellent results in 5 patients, good results in 22, fair in 3 patients with no poor results seen. According to the radiological grading we had excellent results in 4 patients, good in 4 and fair in 3 patients with no poor results Table III.

**Table I:** Patient demographics and data

S. No	Age	Sex	Side	Fracture classification	Fracture type	Mode of injury	Surgical time (minutes)	Time to union (weeks)	Rasmussens score
1	45	M	R	Type V	Closed	RTA	45	11	29
2	34	M	L	Type V	Closed	RTA	50	12	22
3	50	F	L	Type V	Closed	RTA	43	13	24
4	48	M	R	Type VI	Compound	RTA	54	10	27
5	24	F	R	Type V	Closed	FFH	60	11	29
6	60	F	L	Type VI	Closed	RTA	48	12	25
7	54	M	L	Type VI	Closed	RTA	51	10	25
8	38	M	R	Type V	Compound	RTA	54	10	21
9	49	M	R	Type V	Closed	FFH	55	11	24
10	50	F	R	Type V	Closed	RTA	42	12	29
11	29	M	R	Type V	Closed	RTA	44	14	24
12	26	F	R	Type V	Closed	RTA	45	13	26
13	34	F	L	Type VI	Compound	RTA	41	14	25
14	38	M	L	Type V	Closed	RTA	52	11	26
15	51	F	R	Type V	Closed	RTA	60	14	28
16	59	M	L	Type V	Closed	RTA	54	13	25
17	42	M	L	Type V	Closed	RTA	56	14	25
18	47	M	L	Type VI	Compound	FFH	42	15	26
19	54	M	R	Type V	Closed	RTA	49	12	30
20	40	F	R	Type VI	Compound	RTA	50	12	26
21	49	M	R	Type V	Closed	RTA	45	11	24
22	32	M	L	Type VI	Closed	RTA	54	12	26
23	24	M	R	Type VI	Closed	RTA	60	13	24
24	31	M	L	Type V	Compound	RTA	62	14	21
25	40	M	L	Type V	Closed	FFH	58	12	25
26	50	F	L	Type V	Closed	RTA	59	11	26
27	54	M	R	Type V	Closed	FFH	60	11	25
28	38	F	R	Type V	Compound	RTA	54	12	26
29	27	M	R	Type VI	Closed	RTA	48	11	25
30	51	M	R	Type VI	Closed	RTA	45	12	24

Abbreviations: M: Male, F: Female, L: Left, R: Right, RTA: Road traffic accidents, FFH: Fall from height.

**Table II:** Rasmussens functional grading

S. No	Functional grading	No of patients	Percentage
1	Excellent	5	16.66
2	Good	22	73.3
3	Fair	3	10
4	Poor	0	0

**Table III:** Rasmussens radiological grading

S. No	Radiological grading	No of patients	Percentage
1	Excellent	4	13.33
2	Good	23	76.66
3	Fair	3	10
4	Poor	0	0

We had minor complication such as pin tract infection in four patients and superficial infection in two patients which settled down with a course of antibiotics. There were no major complications such as delayed union, nonunion, malunion, deep infection, nerve injury, fracture collapse or wire breakage seen in our study. All patients were satisfied with the functional outcome achieved. None of our patients were lost to follow-up.

## Discussion

Fractures of the proximal tibia are high velocity injuries which are quite difficult to manage. They are often caused by injuries such as road traffic accidents and fall from height. In the elderly age group, they are usually caused by trivial injuries such as a slip and fall due to poor bone stock with advancing age. Schatzkers type V and VI fractures are the most difficult to treat because they are usually associated with considerable soft tissue compromise in the form of compound injuries, deep abrasions, and blister formation. Factors such as communition at the fracture site, varus or valgus displacement, articular depression and associated ligamentous injury with instability further complicate the management. The principles of management in these fractures would be good anatomic reduction, elevation of depressed fragments to restore the integrity of the articular surface, avoidance of valgus or varus angulation, proper soft tissue handling, early mobilisation of the knee to promote a good range of motion and supervised weight bearing^10,11^.

The management options available for these fractures are closed reduction and casting, unilateral external fixation, bicolumnar plating and the hybrid external fixation system. Closed reduction and casting is not advised and is only reserved in patients who are medically unfit for any surgical procedure. A unilateral external fixator is ideally used for temporary immobilisation of the fracture and in compound injuries till the condition of the soft tissues improve to facilitate a definitive internal fixation of the fracture. A longer period of immobilisation with a knee spanning external fixator can cause problems such as knee stiffness with decrease range of motion of the affected knee.

Current literature proposes that the ideal treatment option would be dual plating either through a midline incision or through two separate incisions. This option, however, requires extensive dissection leading to problems with wound healing related to decreased vascularity such as skin and muscle necrosis, increased operative times and as well as having increased risk of infection. These complications can also be encountered even if these fractures are taken up for surgery after a delayed period once the soft tissue status of the limb is in an acceptable condition. The hybrid external fixator eliminates the delay for surgery and hence avoids complications such as knee stiffness, which are quite commonly seen in intra and periarticular fractures of the knee. It is minimally invasive, does not cause problems with soft tissue healing, requires shorter operative times, and is also cost effective to the patient. The hybrid external fixator combines an ilizarov ring with a standard AO frame and can be used as a definitive fixation option in compound injuries as well as in fractures with extensive soft tissue compromise^12,13^.

The fixator acts on the principle of ligamentotaxis to reduce the fracture and to maintain the reduction as well. In bicondylar fractures, the separated or displaced condyles could be compressed with olive wires to provide a stable fixation. In cases of fractures with depression, a mini incision can be made to elevate the depressed fragments without any compromise to the soft tissues. The soft tissue handling is good, there is no blood loss, the fracture haematoma is not disturbed leading to better rates of union and the knee can be mobilised from day one resulting in a good range of motion with a better functional outcome. The fixator is as biomechanically stable as dual column plating^14,15,16^.

The potential disadvantages possible with the hybrid fixator are wire breakage, valgus or varus collapse, pin tract sepsis, septic arthritis if the wires are places within the joint in the capsular reflection less than 10mm from the joint line and common peroneal nerve injury if the wires are passed close to the fibular head. We did not encounter any of these complications in our study. In Aggarwal *et al* study of 56 patients they reported union time of 20.5 weeks with excellent results in 30 patients. They had complications such as osteomyelitis in three patients and pin tract infections in five patients^17^. In Venkatesh G *et al* study of 48 patients the mean union time was 13.3 weeks with 2 cases of delayed union seen. They had excellent results in 8 patients and good results in 30 patients^18^. Savolainan *et al* studied 33 patients and reported a union time of 20 weeks and had complications such as pin tract infection in 7 patients and septic arthritis in 1 patient^19^.

In our study, we only had minor complications such as pin tract infection and superficial skin infections. We had excellent results in 5 patients, good in 22 and fair in 3 patients with no poor results seen. All our patients were happy with the procedure and the final functional outcome. None of our patients were lost to follow-up. We thereby conclude by stating that the hybrid external fixation system is an excellent treatment option in Schatzkers type V and VI fractures associated with soft tissue compromise and gives good functional results.

## Conclusion

The hybrid external fixator combines an Ilizarov ring with a standard AO frame and can be used as a definitive fixation option in compound injuries as well as in fractures with extensive soft tissue compromise. The fixator acts on the principle of ligamentotaxis to reduce the fracture and to maintain the reduction as well. The fixator is as biomechanically stable as dual column plating. The soft tissue handling is good, there is no blood loss, the fracture haematoma is not disturbed leading to better rates of union and the knee can be mobilised from day one resulting in a good range of motion with a better functional outcome.
